# Passivation Characteristics of Alloy Corrosion-Resistant Steel Cr10Mo1 in Simulating Concrete Pore Solutions: Combination Effects of pH and Chloride

**DOI:** 10.3390/ma9090749

**Published:** 2016-09-01

**Authors:** Zhiyong Ai, Wei Sun, Jinyang Jiang, Dan Song, Han Ma, Jianchun Zhang, Danqian Wang

**Affiliations:** 1 School of Materials Science and Engineering, Southeast University, Nanjing 211189, Jiangsu, China; 230139452@seu.edu.cn (Z.A.); sunwei@seu.edu.cn (W.S.); songdancharls@hhu.edu.cn (D.S.); wonderbaba@126.com (D.W.); 2 Jiangsu Key Laboratory of Construction Materials, Nanjing 211189, Jiangsu, China; 3 College of Mechanics and Materials, Hohai University, Nanjing 210098, Jiangsu, China; 4 Research Institute of Jiangsu Shasteel Iron and Steel, Zhangjiagang 215625, Jiangsu, China; mahan-iris@shasteel.cn (H.M.); Zhangjc-iris@shasteel.cn (J.Z.)

**Keywords:** passivation, pH, chloride, corrosion-resistant steel, film composition

## Abstract

The electrochemical behaviour for passivation of new alloy corrosion-resistant steel Cr10Mo1 immersed in alkaline solutions with different pH values (13.3, 12.0, 10.5, and 9.0) and chloride contents (0.2 M and 1.0 M), was investigated by various electrochemical techniques: linear polarization resistance, electrochemical impedance spectroscopy and capacitance measurements. The chemical composition and structure of passive films were determined by XPS. The morphological features and surface composition of the immersed steel were evaluated by SEM together with EDS chemical analysis. The results evidence that pH plays an important role in the passivation of the corrosion-resistant steel and the effect is highly dependent upon the chloride contents. In solutions with low chloride (0.2 M), the corrosion-resistant steel has notably enhanced passivity with pH falling from 13.3 to 9.0, but does conversely when in presence of high chloride (1.0 M). The passive film on the corrosion-resistant steel presents a bilayer structure: an outer layer enriched in Fe oxides and hydroxides, and an inner layer, rich in Cr species. The film composition varies with pH values and chloride contents. As the pH drops, more Cr oxides are enriched in the film while Fe oxides gradually decompose. Increasing chloride promotes Cr oxides and Fe oxides to transform into their hydroxides with little protection, and this is more significant at lower pH (10.5 and 9.0). These changes annotate passivation characteristics of the corrosion-resistant steel in the solutions of different electrolyte.

## 1. Introduction

Most reinforced concrete structures are expected to service for at least 75 years without major repairs [1]. However, this is difficult to achieve, not because of a structural problem but a durability issue. Corrosion of reinforcing steel inside concrete is one of the most important factors that reduce concrete structures durability. In order to minimize or prevent steel corrosion, various methods and techniques [2,3] have been developed and applied, being the most important: concrete cover optimization, electrochemical protection (including cathodic protection, electrochemical realkalization and electrochemical chloride extraction), chemical inhibitors incorporation, epoxy coating on rebar, reinforcement galvanization, etc. However, these mentioned techniques have drawbacks or limitations, failing to prevent steel corrosion of concrete for enough long time.

As one of the most reliable solutions, using “non-corroding” stainless steels is recommended. Some long-term field practice has proved that stainless steel, which provides surpassing chemical resistance, can avoid corrosion problems for a very long time, even in highly aggressive environments [4,5]. However, for their quite high initial costs [6], stainless steels are limited for decades and presently only used in the most critical areas of new structures (such as tidal zones of bridges in maritime regions).

Alloy corrosion-resistant steels, which are more resistant to chlorides or carbonation than plain carbon steel, although less corrosion-resistant than stainless steels but much less costly, have aroused great concern. In recent years, China accelerates the development of independent innovational corrosion-resistant steels. Research Institute of Jiangsu Shasteel Iron and Steel designed and prepared a new alloy corrosion-resistant steel, Cr10Mo1, which has been successfully declared for Chinese invention patents [7]. This steel is alloyed with about 10 wt % Cr and 1 wt % Mo, consisting of granular bainite with ferrite between the grains. Laboratory tests showed that Cr10Mo1 steel has a critical chloride threshold level more than 10 times that of carbon steel, and could be well matched with MMFX steel [8,9], which is one of the most significant and typical corrosion-resistant steels [6], developed by MMFX Steel Corporation, Irvine, CA, USA, in 1998.

Basically, steel reinforcement embedded in concrete forms a thin oxides layer (5~10 nm) in the strong alkaline conditions of concrete pore solution (pH 13~14), referred to as passive film [10], which acts a protective coat and forbids the metal from corroding. For carbon steel, the growth and formation of this passive film is highly affected by both alkalinity and chloride-contamination of the media, as mentioned in many works [11,12]. However, there are limited systematic studies on the combination effects of pH and chloride on the passivation of alloy corrosion-resistant steel and the film surface chemistry.

This work aimed at studying the passive behaviour of Cr10Mo1 alloy corrosion-resistant steel in simulating fresh and carbonated concrete pore solutions with different chloride contents, presenting the combination effects of pH and chloride on the composition and electrochemical characteristics of surface film formed on the steel metal, for a comprehensive understanding of passivation performances of alloy corrosion-resistant steel in different aggressive environments.

## 2. Experimental Procedures

### 2.1. Materials

#### 2.1.1. Test Solutions

According to the reports [13], an alkaline solution with 0.03 M Ca(OH)_2_ (saturated) + 0.2 M KOH + 0.1 M NaOH (pH 13.3), prepared with analytical grade chemicals and Millipore water (18.2 MΩ·cm), was used to simulate the electrolyte in fresh concrete pores. Solutions with pH 12.0, 10.5, and 9.0 were prepared by addition of NaHCO_3_ powder into the 0.03 M Ca(OH)_2_ (saturated) solution (pH about 12.5), to simulate the carbonated or less alkaline concrete pore solutions [14], which could have low pH values even close to 9.0 [15]. The pH of the solutions was carefully checked and monitored throughout by a pH meter (Thermo Scientific Orion pH 2100, Thermo Fisher Scientific, Waltham, MA, USA). The aggressive chloride ions supplied by sodium chloride (also analytical grade) were added into the solutions with the molar concentrations of 0.2 and 1.0 M, which are very often used in corrosion research.

#### 2.1.2. Steel Samples

**Figure 1:**
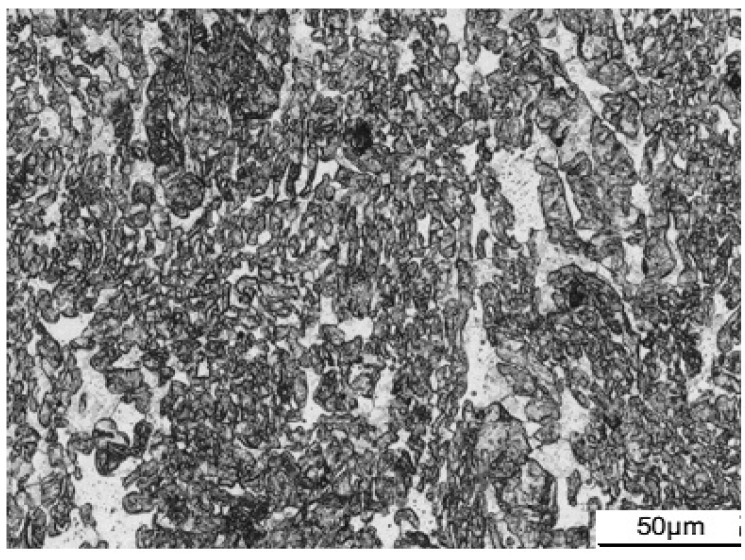
Microstructure of Cr10Mo1 steel obtained by OM.

Experimental materials were alloy corrosion-resistant HRB400 steel Cr10Mo1, designed by Research Institute of Jiangsu Shasteel Iron and Steel. The chemical composition (in wt %) was 0.01% C, 0.49% Si, 1.49% Mn, 0.01% P, 0.01% S, 0.06% V, 10.36% Cr, 1.16% Mo, and the residual Fe. Results of Optical Microscopy (OM) observation of the steel are shown in Figure 1, which indicates granular bainite with ferrite between the grains for the microstructure of the steel.

Steel samples of 10 mm length were cut from ribbed rebars with a diameter of 25 mm. The cross-sections of steel samples were mechanically ground with grades 200, 600, 1000 and 2000 SiC emery papers successively, and polished with alumina paste up to 2.5 μm grit to eliminate the heterogeneities of the steel surface. After polishing, the samples were degreased with alcohol, rinsed with distilled water and dried with a stream of air just before immersion, to ensure their same initial surface states. The samples were kept in test solutions for up to 10 days, during which electrochemical responses were recorded for all the electrodes after 6 h, 1 day, 3 days, 7 days and 10 days immersions, to track the formation process of passive films on the exposed surfaces [16,17].

### 2.2. Electrochemical Measurements

The electrochemical responses of surface films formed on the steel in solutions with different pH and chloride contents were monitored by electrochemical tests. The electrochemical tests, including linear polarization resistance (LPR), electrochemical impedance spectroscopy (EIS) and capacitance measurements (Mott–Shottky approach), were performed at room temperature (25 °C) and under natural aeration, in a classical electrochemical cell with three electrodes, where steel sample as the working electrode was installed with an exposed working area of 1 cm^2^, the reference electrode was a saturated calomel electrode (SCE, all electrode potentials reported in this study were referred to SCE) and a platinum counter electrode was also used.

The LPR measurements were carried out with polarization within ±20 mV from the open-circuit potential (OCP) in the anodic direction with a scan rate of 0.1667 mV/s. The EIS response was recorded following LPR, in a frequency range from 10^4^ Hz down to 10^−2^ Hz with the applied AC amplitude of 10 mV at OCP. Capacitance measurements [18] were performed at a fixed frequency of 1000 Hz (The parameters obtained from Mott–Schottky plots are almost independent when the frequency on the order of kHz is used, according to our previous trials and some references [19]) and a sinusoidal signal of 10 mV, with the polarization applied in successive steps of 50 mV in the cathodic direction from the potential +0.25 V to −1.5 V vs. SCE. There were 3 replicates for each specimen. The equipment used was a PARSTAT 4000 electrochemical system (Princeton Applied Research Inc., Oakridge, TN, USA).

### 2.3. Surface Analysis

Steel samples immersed in the test solutions for 7 d at OCP were withdrawn, rinsed with distilled water and dried with ethanol, and then kept in a vacuum dryer.

The chemical composition and thickness of the surface films on the steel samples were determined by X-ray photoelectron spectroscopy (XPS). A PHI Quantera SXM X-ray photoelectron spectrometer (ULVAC-PHI Inc., Chigasaki, Japan) equipped with a monochromatic Al Kα radiation source (1486.6 eV), a hemispherical electron analyser operating at a pass energy of 55 eV and an analytical chamber with a base pressure of 10^−7^ Pa, was used to collect XPS spectra. The depth profile information was obtained by sputtering the specimens with a scanning argon-ion gun operating at ion energy of 2 keV. The sputtering rate was estimated to be about 0.055 nm·s^−1^. The spectra were calibrated by setting the main line for the C 1s signal of adventitious carbon to 284.6 eV. All XPS spectral analysis was performed by the commercial software XPSpeak version 4.1, which contains the Shirley background subtraction and Gaussian–Lorentzian tail function for better spectra fitting.

The surface morphologies of the samples were examined by scanning electron microscopy (SEM), using a FEI 3D microscope (FEI, Hillsboro, OA, USA) equipped with energy dispersive spectrometer (EDS) microanalysis hardware, which aims to examine the chemical composition of the surfaces.

## 3. Results and Discussion

### 3.1. Surface Film Composition and Structure (XPS Analysis)

**Figure 2:**
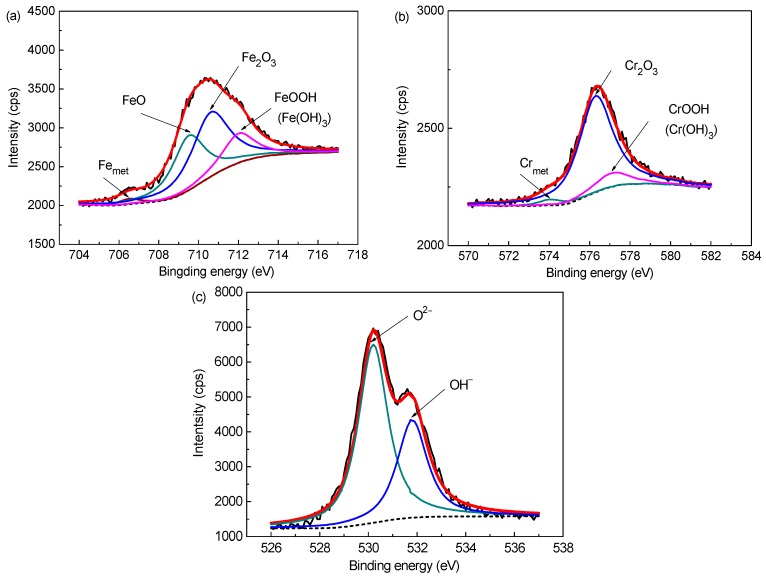
Deconvolution of the Fe 2p_3/2_, Cr 2p_3/2_ and O 1s XPS spectra detected for the passive film on the steel after 7 d immersion in solution of pH 13.3 with 0.2 M Cl^−^: (**a**) Fe 2p_3/2_; (**b**) Cr 2p_3/2_; and (**c**) O 1s.

The survey spectra (not shown) from samples exposed to all media only exhibit signals from the alloy constituents, carbon and oxygen. No traces of components from solution (Na^+^, K^+^, Ca^2+^, and Cl^−^) were detected. The high resolution XPS spectra of Fe-2p, Cr-2p and O-1s signals were deconvoluted into some chemical states which are most probable components needed for corresponding chemical assignments using a deconvolution software XPSpeak version 4.1, based on the average of the binding energies reported in the Handbook of X-ray photoelectron spectroscopy [20] and previous works [21]. According to the deconvoluting results (Figure 2), the Fe 2p_3/2_ signal (Figure 2a) consists of four components, including metallic state (Fe_met_, 706.5 eV), Fe^2+^ in oxide form (FeO, 709.5 eV) and Fe^3+^ in oxide (Fe_2_O_3_, 710.6 eV) and hydroxide forms (FeOOH/Fe(OH)_3_, 712.0 eV). For the Cr 2p_3/2_ spectrum (Figure 2b), there presents three constituent peaks which are assigned to Cr_met_ (573.6 eV), Cr_2_O_3_ (576.3 eV) and CrOOH/Cr(OH)_3_ (577.1 eV), respectively. The peak intensity of the Cr_2_O_3_ is apparently higher than that of CrOOH/Cr(OH)_3_, indicating Cr_2_O_3_ is the dominant Cr species in the passive film for the steel. The O 1s signal (Figure 2c) was fitted using two contributions: one peak at 530.2 eV corresponding to O^2−^ in Fe and Cr oxides, and another one at 531.8 eV corresponding to OH^−^ in hydroxides.

The XPS measurements did not detect the presence of Mo in the passive film on Cr10Mo1 steel; therefore, its performance will not be discussed in this paper, although Mo may produce some considerable effects on electronic properties and electrochemical processes of the passive film, as presented in some works [22].

**Figure 3:**
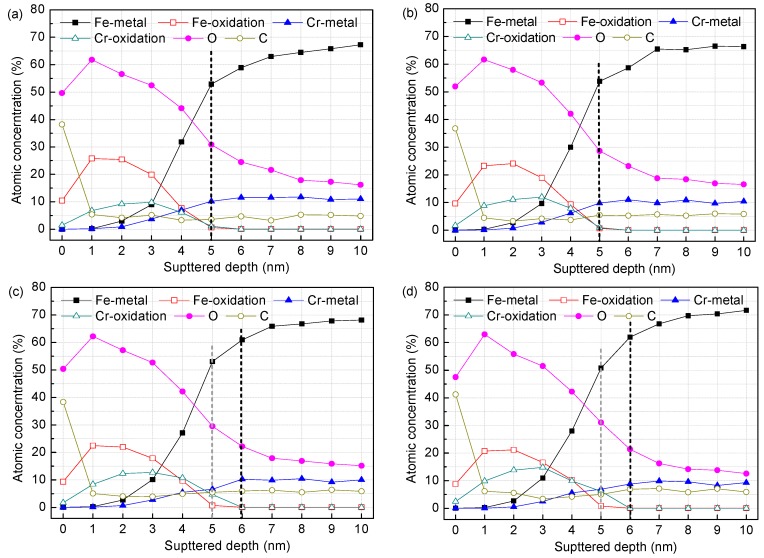
Composition depth profiles obtained from XPS analysis for the surface films on the steel in solutions of different pH with 0.2 M Cl^−^: (**a**) pH 13.3; (**b**) pH 12.0; (**c**) pH 10.5; and (**d**) pH 9.0.

**Figure 4:**
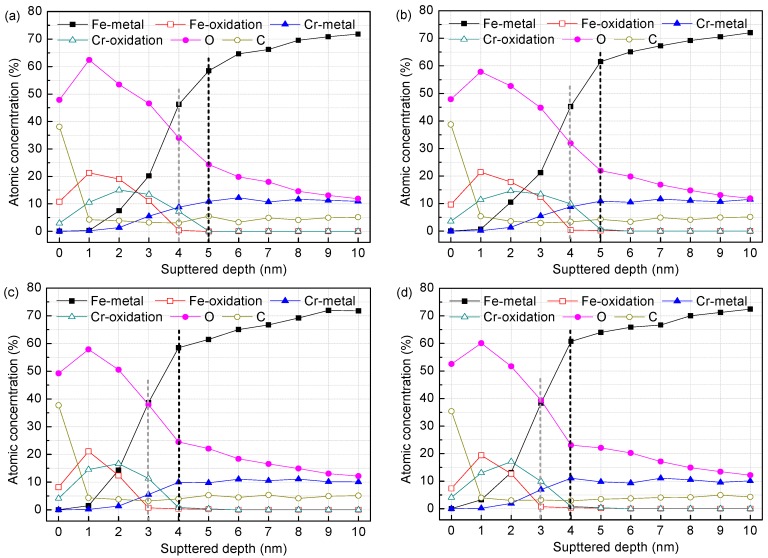
Composition depth profiles obtained from XPS analysis for the surface films on the steel in solutions of different pH with 1.0 M Cl^−^: (**a**) pH 13.3; (**b**) pH 12.0; (**c**) pH 10.5; and (**d**) pH 9.0.

Figure 3 and Figure 4 display the XPS depth profiles of the surface films formed on the corrosion-resistant steel in all test solutions. As can be observed, Fe-metal and Cr-metal concentrations increase progressively with depth and do dramatically where the oxidation state components almost disappear, as expected. This indicates that the predominant metallic state component comes from the substrate when the surface film is sputtered out. The concentration of oxygen, on the other hand, follows an increase initially (about 1~2 nm), and then decreases significantly approaching the metal surface. The high carbon concentration at the surface of each specimen is mainly due to organic carbon contamination (the absorbed alcohol), as referred in other studies [12]. The percentage composition of Fe-oxidation has a sharp rise at first, then reduces gradually and vanishes. Similarly, the Cr-oxidation component also shows an evolution of increasing initially and decreasing afterwards. However, the cut-off points are not identical: for the Fe species the cut-off point is at about 1 nm, while for the Cr species that is at a greater depth of about 2~3 nm. This important difference reveals that the constituents within passive film of the corrosion-resistant steel vary with depth into the layer: the inner region that is adjacent to the metal substrate is a Cr species concentrated layer, while the outer layer is mostly composed of Fe oxides and hydroxides. If Fe-oxidation or Cr-oxidation content tends to zero, it was considered as an interface where the layer is sputtered out [23]. In this term, the surface films on the steel in solutions with 0.2 M chloride are approximately 5 nm, 5 nm, 6 nm and 6 nm thick for pH 13.3, 12.0, 10.5, and 9.0, respectively (Figure 3), while in solutions with 1.0 M chloride are 5 nm, 5 nm, 4 nm and 4 nm thick for pH 13.3, 12.0, 10.5, and 9.0, respectively (Figure 4).

It is noteworthy that, in solutions with 0.2 M Cl^−^, the Cr-oxidation content as a function of pH shows an increasing trend with pH falling, indicating the Cr species have a gradual enrichment in the surface film. Literatures [24,25] attributed this to preferential solubility of Cr/Fe oxides at different pH: at high pH, Cr species become more soluble as chromite ions (CrO^2−^ and CrO_3_^3−^), whereas Fe oxides are more stable; at low pH, the Cr^3+^ concentration within the surface film increases due to the tendency for higher stability of Cr oxides together with the high dissolution of Fe oxides into aqueous solution when the pH drops. However, the Cr species enrichment at low pH cannot be explained just in terms of Fe oxides relative decreasing resulted by selective dissolution. It is most probably the consequence of the further formation and growth of Cr oxides, because the Cr-concentrated inner layer in the surface film becomes more thicken when pH drops from 13.3 to 9.0 (Figure 3), which might result from excessive dissolution of Fe and Cr from the substrate. When exposed to less alkaline media, Fe oxides in the outer layer decompose gradually and release more soluble ions into solution, promoting metallic Fe beneath the film to dissolve into the film layer as Fe(OH)_aq_ [26]. Although lower pH facilitates dissolution of the excessive Fe, little new Fe species form in the film layer for the high dissolution rate of Fe oxides at the film/solution interface, so the Fe-based outer layer has no growth, as illustrated in Figure 3. Following metallic Fe dissolution, Cr from the metal also dissolves excessively and produces new Cr oxides due to the tendency for its very high stability. The newly formed Cr species precipitate in the inner layer and therefore the thickness of the Cr-oxides layer experiences an increase. Freire et al. [18] investigated the effect of pH on passive behaviour of AISI 316 stainless steel in alkaline media in the absence of chloride, and recognized that Cr oxides has an increasing enrichment accompanied by the passive film thickening at lower pH. It should be stated that this is also the same case for Cr10Mo1 corrosion-resistant steel, at least if the environment is moderately contaminated by chloride. When the chloride reaches 1.0 M (Figure 4), there is still a gradual enrichment of the Cr species in the surface film following pH decreasing, however, the film exhibits important changes, i.e., as pH falls below 10.5, the thickness of the film suffers a decline, coming to 4 nm, in contrast with 6 nm thickness of the layer in the presence of 0.2 M Cl^−^. This indicates that increasing chloride has some negativity on the passivation of the steel and this effect is more obvious in low pH (10.5 and 9.0) media.

**Figure 5:**
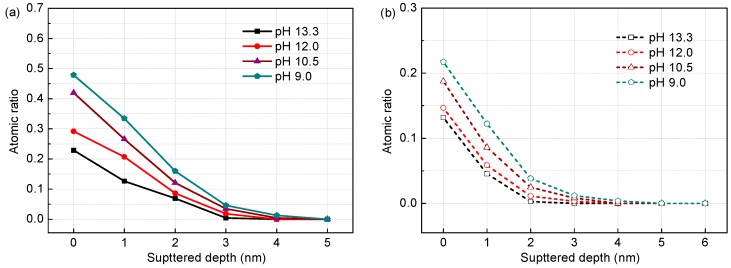
Composition depth profiles obtained from XPS analysis for the surface films formed on the steel in solutions of different pH with 0.2 M Cl^−^: (**a**) Fe_hy_/Fe_ox_; and (**b**) Cr_hy_/Cr_ox_.

**Figure 6:**
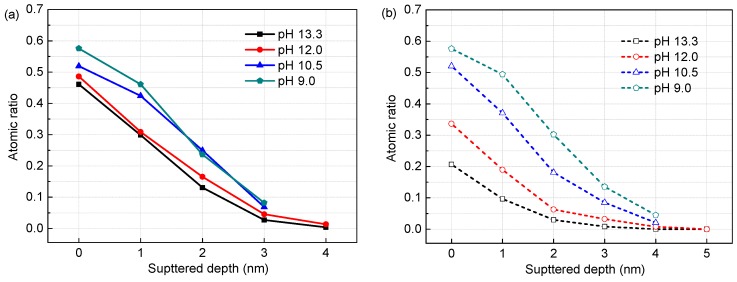
Composition depth profiles obtained from XPS analysis for the surface films formed on the steel in solutions of different pH with 1.0 M Cl^−^: (**a**) Fe_hy_/Fe_ox_; and (**b**) Cr_hy_/Cr_ox_.

Figure 5 and Figure 6 show the composition profiles (for atomic ratios of Fe_hy_/Fe_ox_ and Cr_hy_/Cr_ox_ at various sputtered depths) of the surface films on the corrosion-resistant steel in different test solutions, obtained from quantitative XPS analysis according to the peak intensity of components. Generally, with increasing sputtered depth, the contents of Fe and Cr hydroxides decrease gradually and disappear finally, indicating the hydroxides are the predominant components near the free surface of the film. It can be observed that with the decreasing of pH, the Fe_hy_/Fe_ox_ ratio has a continuous rise at the same depth into the film (Figure 5a and Figure 6a). In fact, less alkaline media favours Fe hydroxides formation [26]. When the surface film is exposed to lower pH, FeO (Fe_3_O_4_) and Fe_2_O_3_ decompose gradually, converting into porous and loose FeOOH/Fe(OH)_3_. This induces the Fe-oxides layer becomes more and more defective. From comparison of the Fe_hy_/Fe_ox_ in Figure 5a and Figure 6a for an identical pH, increasing chloride also brings about some rise of the Fe_hy_/Fe_ox_ ratio at the same depth into the film, indicating a similar effect to that of decreasing pH. This is expected, because Cl^−^ favours adsorbing on a passive film and then occupying oxygen vacancies by taking place of OH^−^ in the oxides. The continuous increase in the vacancy production induces the formation of cavities in the oxides, eventually leading to the oxides destruction [27]. Thus, both carbonation and chloride could cause damage of the Fe-oxides layer and therefore reduce its protection, as mentioned in references [26,28]. Similar to the Fe_hy_ content evolution, Cr_hy_ concentration in Cr species also has an increasing trend with pH falling (Figure 5b and Figure 6b), but the change is dependent on chloride contents. In solutions with 0.2 M Cl^−^, the Cr_hy_/Cr_ox_ value remains small, at levels below 0.25 (Figure 5b), indicating most of Cr oxides maintain intact. However, with Cl^−^ content increasing to 1.0 M, this ratio at the film surface enhances sharply from 0.24 to 0.58 when pH drops from 13.3 to 9.0 (Figure 6b), meaning high chloride promotes the substantial formation of Cr hydroxides at low pH, which are also low protective [29].

**Figure 7:**
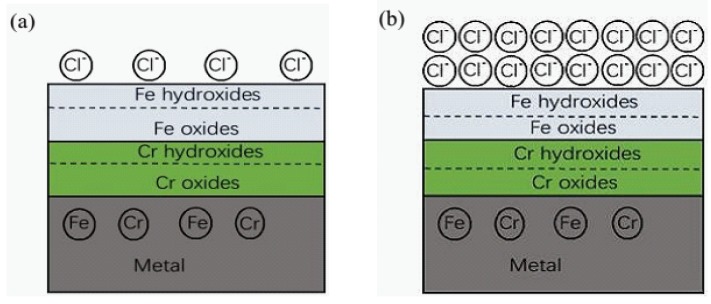

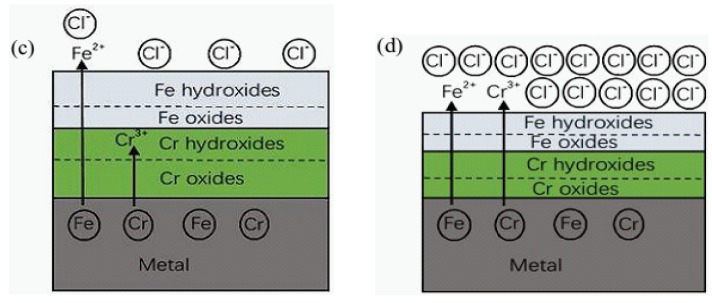
Schematic illustration of the growth processes of passive films on alloy corrosion-resistant steel Cr10Mo1 in solutions with different pH and Cl^−^ contents: (**a**) with pH 13.3 and 0.2 M Cl^−^; (**b**) with pH 13.3 and 1.0 M Cl^−^; (**c**) with pH 9.0 and 0.2 M Cl^−^; and (**d**) with pH 9.0 and 1.0 M Cl^−^.

Figure 7 presents the growth processes of passive films on the steel in solutions of different pH with chloride contents. The thermodynamic stability of oxides or hydroxides, which is affected by the concentrations of hydroxyl ions and chloride ions [30], determines passive film formation and growth, as confirmed by the results obtained by XPS analysis in the present study. The following mechanism for passive film formation and growth on Cr-series stainless steels in alkaline solutions has been proposed in published works [31,32]. When the alloy is exposed to an alkaline electrolyte, Fe would prefer to dissolve and faster diffuse into the solution and therefore be enriched at the film/solution interface while dilatory dissolving and slower diffusing component like Cr will remains in the region nearer the metal, with little movement. Contacting with OH^−^ predominantly, dissolved Fe/Cr initially forms hydroxides. With increasing passivation time, the hydroxides will dehydrate into oxides and then the oxides layer grows gradually.

(1) Fe^2+^ + 2OH^−^ → Fe(OH)_2_↓

(2) 4Fe(OH)_2_ + O_2_ + 2H_2_O → 4Fe(OH)_3_

(3) 2Fe(OH)_3_ → Fe_2_O_3_ + 3H_2_O

(4) 2Fe(OH)_3_ + Fe + 2OH^−^ → Fe_3_O_4_ + 4H_2_O + 2e^−^

(5) Cr^3+^ + 3OH^−^ → Cr(OH)_3_↓

(6) Cr(OH)_3_ + Cr + 3OH^−^ → Cr_2_O_3_ + 3H_2_O + 3e^−^

The outer Fe-oxides layer grows by the diffusion of metallic ions through micropores in the film and the inner Cr-oxides layer grows by access of OH^−^ to the film/alloy interface through the micropores in the film. The continuously growing oxides layer will block the transport of metallic ions and hydroxyl ions and further slow down the growth of itself. As a result, the passive film is constructed by a Cr oxides and hydroxides concentrated inner layer (grown from alloy matrix) and an outer layer enriched in Fe oxides and hydroxides (formed by a diffusion and precipitation process). It should be noted that in solutions of high pH (13.3), high chloride not more than 1.0 M depresses little the growth and formation of the passive film on the corrosion-resistant steel (Figure 7a,b). When in solutions of low pH (9.0), metallic Fe/Cr dissolves and diffuses faster, but the precipitation of the hydroxides and oxides, especially Fe hydroxides and oxides, become more difficult for the diluted OH^−^ concentration. This would allow the excessive dissolution of the Fe and Cr, in another vein, more metallic Fe and Cr dissolve from the metal. Thus, the inner Cr-oxides layer has further growth and becomes more thicken, but the outer Fe-species layer is not in the same case, due to its very high solubility at low pH (Figure 7c). However, in the presence of 1.0 M chloride, the inner Cr-oxides layer has no growth as in the presence of 0.2 M chloride, but decreases dramatically in the layer thickness, together with further reduction of the Fe-species outer layer (Figure 7d), due to the standard free energies of Fe and Cr oxides in more severe environments are substantially enhanced [30].

### 3.2. Electrochemical Results

#### 3.2.1. Capacitance Measurements (Mott–Schottky Plots)

The semi-conductive behaviour and electronic properties of the passive oxides film can be assessed by measuring the capacitance of the electrode/electrolyte interface. When the oxides film is in contact with an electrolyte, the capacitance of the electrode/electrolyte interface (C) can be described by the capacitance of space charge layer (C_sc_) and the capacitance of the Helmholtz layer (C_H_) as two condensators in series, neglecting the Guoy–Chapman diffuse layer and surface states contribution for the total capacitance [33]. When the used frequency is high enough (on the order of kHz), the measured C is considered to be approximately equal to the C_sc_, for the C_H_ contribution could be negligible [34]. Assuming that space charge layer of the semiconductor is under depletion conditions, the determined capacitance of electrode/electrolyte interface, C, and the applied potential, E, can be described by Mott–Schottky relationship:

for n-type semiconductor
  (7) $$C^{-2}=\frac{2}{{\varepsilon \varepsilon }_{0}eN_{d}}\left(E-E_{\text{FB}}-\frac{\text{KT}}{e}\right)$$
for p-type semiconductor
  (8) $$C^{-2}=-\frac{2}{{\varepsilon \varepsilon }_{0}eN_{a}}\left(E-E_{\text{FB}}+\frac{\text{KT}}{e}\right)$$

where ε is the dielectric constant of the semiconductor (usually taken as 15.6 [33,35] for the oxides films formed on alloys), ε_0_ is the vacuum permittivity (ε_0_ = 8.85 × 10^−14^ F·cm^−1^), N_d_ and N_a_ are the donor density and acceptor density, respectively, e is the elementary charge (e = 1.602 × 10^−19^ C), k is the Boltzmann constant (k = 1.38 × 10^−23^ J·K^−1^), T is the absolute temperature, and E_FB_ the flat band potential. The KT/e term can be neglected as it is only about 25 mV at room temperature. The carrier density can be calculated from the slope of the experimental C^−2^ versus E plot (a negative slope is for a p-type semiconductor response inversely proportional to the acceptor density N_a_, while a positive slope for a n-type semiconductor also inversely proportional to the donor density N_d_).

**Figure 8:**
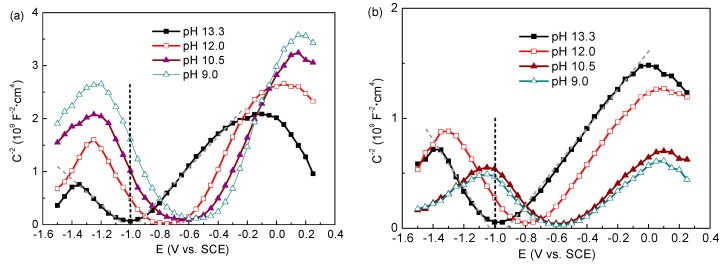
Mott-Shottky plots for passive films formed on the steel in solutions with different pH and Cl^−^ contents at 7 d immersion: (**a**) 0.2 M; and (**b**) 1.0 M.

Figure 8 displays the Mott–Schottky plots recorded for the passive films formed on the steel after 7 d immersion in all test solutions. It can be observed that in the Mott–Schottky plots two linear regions are presented, indicating the passive film formed on the steel exhibits both n-type (positive slope) and p-type (negative slope) semiconducting characteristics irrespective of the exposure conditions. The duplex semiconductor character of the passive film is related to its chemical composition. Its n-type semiconductor behaviour can be attributed to Fe oxides and hydroxides, and p-type semiconductor behaviour to Cr species, according to previous investigations [33,35].

In Table 1, the carrier concentrations (donor and acceptor species, N_d_ and N_a_, respectively) for the semiconductor passive films are listed. In solutions of 0.2 M Cl^−^, the steel has declined carrier concentrations as pH varies from 13.3 to 9.0, meaning the film behaves as a semiconductor with poorer and poorer electrical conductivity. This evolution indicates a more effective corrosion protection of the surface film at lower pH. According to the point defect model (PDM) proposed by Macdonald and co-workers [36], the passive film contains a number of point defects, such as oxygen and/or cation vacancies, which act as donors and acceptors, respectively. Carbonation promotes the excessive dissolution of Fe and Cr from the metal and results in more Fe and Cr species formed in the film, which have decreased oxygen and cation vacancy concentrations. However, in test solutions with 1.0 M Cl^−^, this trend is reversed. N_a_ and N_d_ of the steel have far higher values at pH 9.0 than that at pH 13.3. This substantial change in carrier concentrations corresponds to non-stoichiometry defects in the space charge region or disordered character of the surface film [37]. Thus, the surface film becomes more and more defective with pH decreasing, and this is associated with the gradual destruction of the protective oxides, as shown by XPS measurements.

#### 3.2.2. Linear Polarization Resistance

Linear polarization resistance (LPR) monitoring is a non-destructive technique for measuring the corrosion rate of reinforcing steel and accurately evaluating its condition, which has been discussed in detail in many works [38]. From LPR tests, the corrosion potential, E_corr_, which directly indicates the corrosion state, and polarization resistance, R_p_, which is related to the corrosion rate of a corrosion process, can be obtained directly by the built-in fitting software.

**Figure 9:**
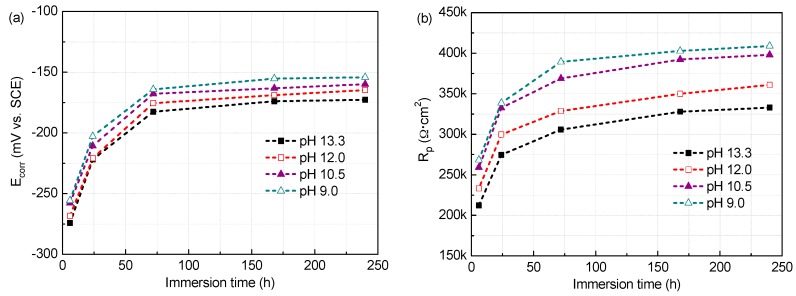
The corrosion potential (E_corr_) and polarization resistance (R_p_) of the steel as a function of time in solutions of different pH with 0.2 M Cl^−^: (**a**) E_corr_; and (**b**) R_p_.

**Figure 10:**
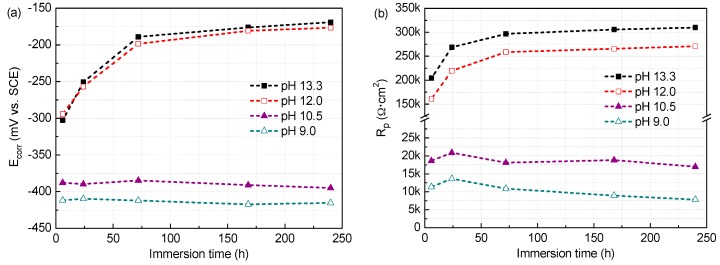
The corrosion potential (E_corr_) and polarization resistance (R_p_) of the steel as a function of time in solutions of different pH with 1.0 M Cl^−^: (**a**) E_corr_; and (**b**) R_p_.

The E_corr_ and R_p_ values of the steel in all test solutions against time (6 h, 1 day, 3 days, 7 days, and 10 days) are presented in Figure 9 and Figure 10. The results show that both E_corr_ and R_p_ values are affected by the exposition conditions. In solutions with 0.2 M Cl^−^, E_corr_ values of the steel get continuous increasing and reach above −200 mV after 3 d immersion, with variations from −200 to −150 mV vs. SCE, indicating complete passivity of the steel at all the pH values [39]. However, when the Cl^−^ content rises to 1.0 M, high E_corr_ values above −200 mV could stay up only when at pH 13.3 and 12.0. At pH below 10.5, the E_corr_ values have no significant change from the beginning of immersion, and show slight rise and fall over the immersion time, but remains below −350 mV, suggesting difficult passivating of the steel.

At early immersion, the R_p_ is relatively small, and then increases gradually and tends to remain stable after 7 d immersion. This behaviour should be attributed to the fact that at the beginning of the immersion, the surface of the steel sample is active and consequently the R_p_ is low, but as the exposure time increasing, the surface of the steel sample is covered by a corrosion products film, and consequently the R_p_ enhances and gets to stable feature until basically mature passive film is formed (after processing for 7 days). Certainly, this trend is in cases except for low pH (10.5 and 9.0) with 1.0 M Cl^−^. Generally, at the same pH, R_p_ values obtained in 0.2 M Cl^−^ condition are higher than that in solutions containing 1.0 M Cl^−^. The higher the R_p_ value, the stronger the corrosion prevention capability of the surface film [40]. So chloride reduces the passivity for the steel and this is more significant at low pH. It is noteworthy that the R_p_ has different evolution with the pH varying, depending on the chloride contents. In solutions with 0.2 M Cl^−^, R_p_ values have marked raise at lower pH, and almost show an increment of 1/4 when pH drops from 13.3 to 9.0. However, with exposure to high chloride (1.0 M), R_p_ decreases prominently with pH. When pH is below 10.5, R_p_ fluctuates in the range of 5~20 kΩ·cm^2^, which are very low values, with little enhancement even if somewhat decline after 7 d immersion, revealing the steel hardly passivates for the surface films are very electric conductive, in consistent with Mott–Schottky plots analysis.

#### 3.2.3. Electrochemical Impedance Spectroscopy

**Figure 11:**
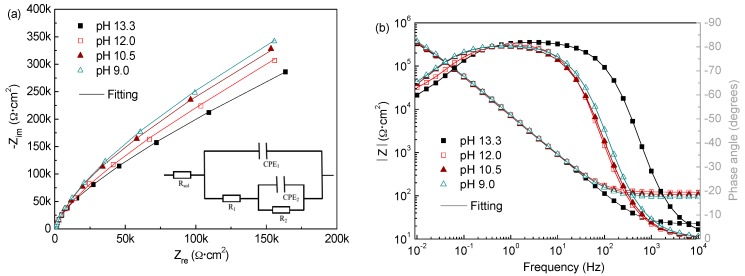
Measured EIS (in Nyquist and Bode forms) of the steel in solutions of different pH with 0.2 M Cl^−^ after 7 d immersion: (**a**) Nyquist plots; and (**b**) Bode plots.

**Figure 12:**
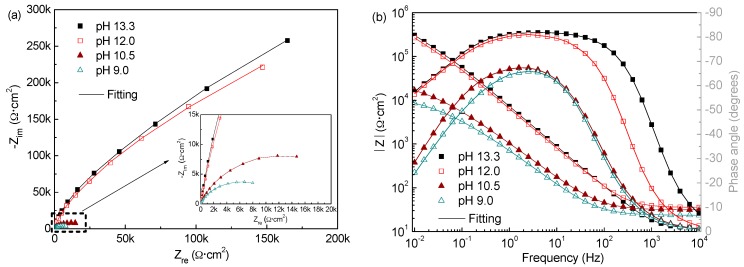
Measured EIS (in Nyquist and Bode forms) of the steel in solutions of different pH with 1.0 M Cl^−^ after 7 d immersion: (**a**) Nyquist plots; and (**b**) Bode plots.

Figure 11 and Figure 12 show the EIS spectra in Nyquist and Bode forms obtained for the corrosion-resistant steel after 7 d immersion in solutions of pH from 13.3 to 9.0 with different Cl^−^ contents. It is evident that the EIS spectra profiles of the steel with the pH varying for the two Cl^−^ contents are materially different. In solutions with 0.2 M Cl^−^, the steel shows capacitive-like behaviour with the maximum phase angles close to −90° and high values of |Z| above 300 kΩ·cm^2^ in the region of low frequencies, with regard to the Bode plots, suggesting that the passive films formed on the steel at all pH offer high corrosion resistance. It is noted that the impedance response is increasing following the pH dropping, and the capacitive arc radius and overall impedance are even larger at pH 9.0, indicating the film formed at lower pH exhibits more protective behaviour. In contrast, when chloride rises to 1.0 M, the capacitive arc radius markedly decreases as the pH does. The overall impedance has very low values in the order of 10~20 kΩ·cm^2^ when the pH falls below 10.5, signifying that the steel hardly passivates in low pH media. These results are in good agreement with the Mott–Schottky approach and LPR observation. Indeed, this change is ascribed to the chemical composition evolution of the surface film formed on the steel with pH and Cl^−^ contents varying, as mentioned above.

The Zsimpwin program was used to fit the EIS data. Based on some trials and references [18,23], the equivalent circuit depicted in Figure 11a was adopted to fit the experimental data, which provided a right fitting with errors within 10%. The constant phase element (CPE) is used to descript the frequency dispersion behaviour of non-ideal capacitors with its impedance (Z_CPE_) defined by Equation (9)

(9) $$Z_{\text{CPE}}=\frac{1}{Y_{0}{\left(\text{jw}\right)}^{n}}$$

where Y_0_ is the CPE electrical constant admittance, ω is the angular frequency (in rad/s), j is the imaginary number (j^2^ = −1) and n is the CPE exponent, as an adjustable parameter affected by non-homogeneities and roughness of the surfaces, that always lies from 0 to 1.

For the meaning of the circuit elements in this circuit model, the following physical interpretation is adopted [18,23]: the resistance connected in series with the two time constants corresponds to the ohmic resistance of the solution (R_sol_), which changes with ion concentrations of the test solution. The high frequency time constant (R_1_, CPE_1_) can be attributed to the charge transfer processes in the active surface areas (film defects/pores) and it is represented by the charge transfer resistance (R_1_) coupled with the double layer capacitance (simulated by CPE_1_). The low frequency time constant (R_2_, CPE_2_) was assigned to the redox processes taking place in the areas covered with the passive film (protective oxides) and it is composed of the passive layer resistance (R_2_) and the passive film capacitance (CPE_2_).

Table 2 presents the fitting parameters obtained from the experimental EIS spectra of the steel in all test solutions at 7 d immersion time. It can be observed that in presence of 0.2 M Cl^−^, both R_1_ and R_2_ have the highest values at pH 9.0. This is obviously ascribed to the further growth and formation of protective Cr oxides on the metal when pH decreasing, which provides higher resistance to the corrosion processes as proposed in literatures [18]. The admittance evolution of CPE_2_ agrees well with that of R_2_. It varies in the range 2.1 × 10^−5^~1.7 × 10^−5^ Ω^−1^·cm^−2^·s^n^ as the pH drops from 13.3 to 9.0, reflecting the passive film capacitance behaviour are somewhat more significant at lower pH. However, the CPE_1_ values show an upward tendency as the media becomes less alkaline, suggesting that the dispersion effect of the double layer capacitance becomes more highlighted. This reveals the overall film surface becomes rougher and more heterogeneous, for more Fe hydroxides with porous and loose structure form in the outer layer at lower pH. Even so, the film formed on the steel at lower pH has greater protection, as evidenced by the higher R_1_ and R_2_ values, in agree well with the LPR and Mott–Schottky analysis results. On the contrary, in the presence of 1.0 M Cl^−^, R_1_ and R_2_ suffer an important drop as pH, and exhibit extremely low values less than 10 kΩ·cm^2^ at pH 9.0, suggesting the very fast electrochemical corrosion process. Certainly, this is related with the formation of a surface film with more defects. CPE_1_ increases about one order of magnitude and CPE_2_ exhibits a similar trend when pH below 10.5, indicating the steel more approachable to accomplish depolarization [41]. All these signify that the film becomes less and less protective owing to its deterioration.

### 3.3. Surface Morphology

**Figure 13:**
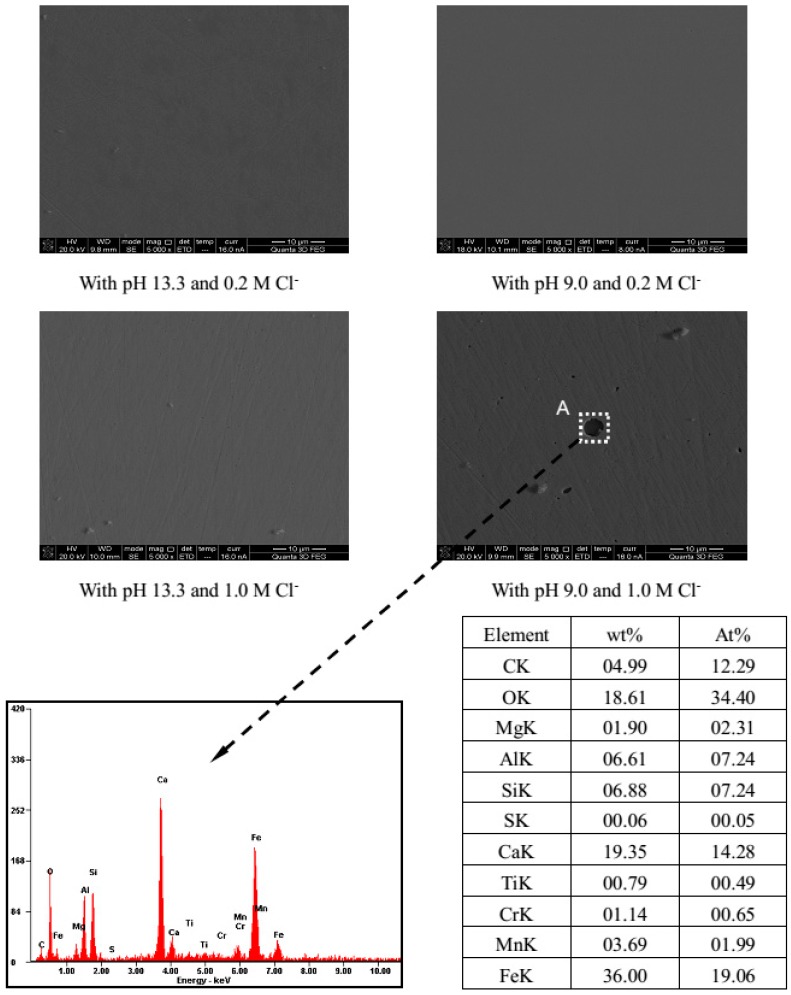
SEM images of the steel surfaces immersed for 7 d in solutions with different pH and Cl^−^ contents, and EDS spectra registered on the characteristic areas of SEM images.

Figure 13 presents several representative images of the steel samples in different exposure conditions. It is observable that, in presence of 0.2 M Cl^−^, the SEM images show a clean and bright steel surface for all the pH values, with almost no spots. When exposed to 1.0 M Cl^−^, the steel has the same case as above for pH 13.3, but for low pH values (pH 9.0), some pits rendering black dish-like holes were observed on the metal surface, and different attack morphologies could also be found. Selecting typical black dish-like holes as areas (e.g., area A) for EDS chemical analysis. The results reveal that the composition of chemical species inside the area A includes about iron (36.0%), calcium (19.4%), chromium (1.1%), oxygen (18.6%), and also small amounts of other inclusion elements from the metal (The considerable amounts of Al (6.61%) may originate from alumina paste adhering to the surface of steel when the steel is polished). This indicates some corrosion products (as Fe/Cr hydroxides) and calcium hydroxide crystals (from the solution) attached into the pitting hole, suggesting the area has suffered some corrosion degree.

## 4. Conclusions

The passive behaviour of alloy corrosion-resistant steel Cr10Mo1 in simulating concrete pore solutions with different pH values (from 13.3 to 9.0) and chloride contents (0.2 M and 1.0 M) was investigated. Analytical and electrochemical results proved that the exposure conditions modify the chemical composition and electrochemical responses of the surface film.

Surface composition analysis performed by XPS revealed that the passive film formed on the corrosion-resistant steel consists of Fe and Cr oxides/hydroxides, which presents in two layers with the outer layer mainly composed of Fe oxides and hydroxides, and the inner one enriched with Cr species. In presence of 0.2 M chloride, as the pH drops, Fe oxides in the outer layer become more soluble but Cr oxides in the inner layer maintain good stability and have further growth, on account of excessive dissolution of metallic Fe and Cr from the substrate promoted by lower pH. However, in presence of 1.0 M chloride, both the Fe-oxides outer layer and Cr-oxides inner layer suffer a significant decrease in thickness, and substantial Fe and Cr hydroxides form in the surface film when pH drops below 10.5, indicating high chloride produce much negative effect on the passive film formation of the steel in low pH (10.5 and 9.0) media.

Mott–Schottky analysis suggests the passive film performs n-type and p-type semiconductor behaviour related to its bilayer structure composed of Fe and Cr species. In presence of 0.2 M chloride, the surface film has poorer electrical conductivity with pH dropping mainly due to Cr oxides with excellent protection get enriched in the film. However, high chloride (1.0 M) facilitates Cr oxides to convert into their hydroxides which are defective, and the case is reversed: the lower the pH value, the more electric conductive the film.

LRP and EIS tests evidence that, in solutions with relatively moderate chloride (0.2 M), Cr10Mo1 steel gets good and stable passivity after 7 d immersion no matter the pH drops, and in view of the electrochemical responses, lower pH provides better conditions for the steel passivation, due to more protective Cr oxides forming and precipitating in the inner layer. However, in presence of high chloride (1.0 M), there is an inversion of this trend. The passivity is dramatically weakened with increasing carbonation, and even pits occur when pH is below 10.5 as shown by SEM/EDS analysis. This can be related to the major decomposition of protective Fe and Cr oxides, which induces the formation of a defective and porous surface layer rich in hydroxides.

## Figures and Tables

**Table 1:** Effect of pH values and chloride contents on semiconducting properties of passive films formed on the steel.

pH	0.2 M		1.0 M	
	N_d_ (10^20^ cm^−3^)	N_a_ (10^20^ cm^−3^)	N_d_ (10^20^ cm^−3^)	N_a_ (10^20^ cm^−3^)
13.3	29.65	36.53	49.11	41.26
12.0	18.21	19.72	45.90	43.47
10.5	16.41	14.61	59.51	68.28
9.0	15.98	12.54	63.26	78.02

**Table 2:** Best fitting parameters for the experimental EIS of the steel in test solutions with different pH and Cl^−^ contents after 7 d immersion.

Cl^−^ Contents (M)	pH	R_sol_ (Ω·cm^2^)	R_1_ (Ω·cm^2^)	CPE_1_		R_2_ (Ω·cm^2^)	CPE_2_	
				Y_0_ (Ω^−1^·cm^−2^·s^n^)	n		Y_0_ (Ω^−1^·cm^−2^·s^n^)	n
0.2	13.3	18.2	3.54 × 10^5^	2.47 × 10^−5^	0.92	11.37 × 10^5^	2.07 × 10^−5^	0.82
	12.0	121.2	4.93 × 10^5^	2.54 × 10^−5^	0.91	13.23 × 10^5^	1.94 × 10^−5^	0.82
	10.5	106.3	6.84 × 10^5^	2.75 × 10^−5^	0.90	15.62 × 10^5^	1.85 × 10^−5^	0.80
	9.0	96.8	7.31 × 10^5^	2.87 × 10^−5^	0.89	17.36 × 10^5^	1.73 × 10^−5^	0.80
1.0	13.3	11.8	2.96 × 10^5^	2.58 × 10^−5^	0.91	8.85 × 10^5^	2.23 × 10^−5^	0.81
	12.0	35.7	2.31 × 10^5^	2.91 × 10^−5^	0.90	6.13 × 10^5^	2.07 × 10^−5^	0.81
	10.5	30.4	1.06 × 10^4^	2.12 × 10^−4^	0.81	1.89 × 10^4^	1.63 × 10^−4^	0.59
	9.0	23.2	3.21 × 10^3^	2.93 × 10^−4^	0.79	9.76 × 10^3^	2.41 × 10^−4^	0.53
